# Primordial radionuclides in the dust samples from the educational institutions of central Bangladesh: radiological risk assessment

**DOI:** 10.1016/j.heliyon.2022.e11446

**Published:** 2022-11-07

**Authors:** Md. Joynal Abedin, Rahat Khan

**Affiliations:** a Centre for Higher Studies and Research, Bangladesh University of Professionals (BUP), Mirpur Cantonment, Mirpur, Dhaka 1216, Bangladesh; b Institute of Nuclear Science & Technology, Bangladesh Atomic Energy Commission, Savar, Dhaka 1349, Bangladesh

**Keywords:** Classroom-dust samples, Primordial radionuclides (^226^Ra, ^232^Th, ^40^K), Educational institutions, Environmental distributions, Radiological health hazards

## Abstract

For the first time, this study presents the radio-activity concentrations of primordial radionuclides in a suite of classroom-dusts collected from 23 schools in central part of Bangladesh. Bulk elemental compositions from instrumental neutron activation analysis (INAA) were transformed into accompanied radio-activity contents (Bq.kg^−1^). Mean activity contents of ^226^Ra, ^232^Th, & ^40^K in dust samples were 86.0, 43.4, and 448 Bq.kg^−1^, respectively, which were comparatively elevated relative to the relevant world average value. Higher NORMs abundances were due to the surface soil weathering and aerodynamic fractionations. Estimation of typical radiological-risk indices demonstrates human health risks. Bearing in mind that the greater susceptibility of school-going juveniles & children to the ionizing-radiations & the entering of NORMs-comprising dust-particle into human lungs, calculated radiological indices merely represent the least potential risk. However, in actual cases, α-particles from the ^238^U and ^232^Th-decay series can create significant radiation-damage to the respiratory-system.

## 1 Introduction

Naturally occurring primordial radioactive nuclides in ambient environment are of great concern for their hazardous impacts on human-health through ionizing radiations. Various types of physiological problems, e.g., lung cancer, renal failure, kidney dysfunction, and bone deformities may be caused by exposure to NORMs (Majlis et al., 2022; Islam et al., 2022; Habib et al., 2019a, 2019b). NORMs distributions in differential environmental constituents (e.g., dust, sediment, soil, water) mostly depend on climatic conditions, local geology, & weathering processes (Khan et al., 2022a, 2022b; Alonso-Hernández et al., 2018; Alam et al., 1999). NORMs' occurrence in soil and/or sediment is mostly correlated with external exposure to radiation if the inhalation of gaseous radon is overlooked. Though the NORMs' impacts by water having various exposure routes, the impacts seem trivial due to the very low abundances of NORMs in the natural resources of water (Habib and Khan 2021; Arogunjo et al., 2009). Moreover, like water, radioactivity abundance in dust cannot be disregarded. Additionally, like soil or sediment, exposures of radiation through dust-NORMs are not limited merely through external-route (ignoring the inhalation of radon). Rather dust can have the probability of entering in lungs through the inhalation route (Apeagyei et al., 2011; Ahmad and MatiullahKhatibeh, 1997) along with its radionuclides.

In urban areas, dust particles normally originating from surface soil through several weathering progressions, e.g., collective actions of heat and turbulent airflow, rain, water logging, etc. Other than the anthropogenic processes, natural processes, e.g., construction works, industrial activities, vehicular transportation, etc. play significant role in the formation of dust and determine the compositions of elements in the generated dust (Addo et al., 2020). Mostly dust particle generated from surface geo-materials (e.g., soil or/and sand), which coherently carry NORMs in it’s compositions, can eventually cause radiological risks. Dust, particularly road-dusts have been studied by numerous researchers (e.g., Ahmed et al., 2007; Ghanavati et al., 2018; Abbasi et al., 2013; Mohtaderi et al., 2019; Kormoker et al., 2021; Kabir et al., 2021a, 2021b) for potentially toxic heavy metal’s abundances. However, research on NORMs concentrations in dust samples have hardly been conducted (Addo et al., 2020; Abedin and Khan, 2022; Mahdi et al., 2015). Moreover, classroom-dusts collected from different educational institutions have rarely been conducted, even for elements having potential toxicity. Bulk chemical compositions of indoor-dusts can also be correlated to the room’s air-quality (Praveena et al., 2015; Soltani et al., 2015) that may also cause health risks. Furthermore, children & juveniles are relatively more vulnerable to environmental heavy metal(oid)s (Habib et al., 2020; Jannat et al., 2022; Mohtaderi et al., 2019; Islam et al., 2020a, 2020b; Medunic et al., 2016; Kumar et al., 2022, 2021a, 2021b; Ahmed et al., 2021; Hossain et al., 2022a, 2022b; Rahman et al., 2022; Anik et al., 2022). Thus, studies on NORM’s occurrence in the dust of classrooms are of utter importance. But the literature studies on the NORM’s abundance in classroom dust are too scarce to obtain.

Measurements of NORMs abundances have normally been carried out by HPGe-detector which requires 100–250g solid samples. Moreover, samples required at least 28 days to attain the secular equilibrium (between short-lived daughter & long-lived parent) followed by a long time γ-ray-counting (25,000–50,000s) (Bramha et al., 2018; Ehsan et al., 2019). If the mass of samples is lower, it requires a longer counting time. Additionally, higher uncertainty is also introduced from background radiations in measured data. However, collected mass of dusts from the surface of the classroom benches are typically low (∼20–25g), it does not permit for determining the primordial radionuclide’s activity conventionally (described before). So, a passive way has been adopted to measure NORMs in the dust samples to overcome this problem. In this study, a precise and highly accurate, non-destructive and primary nuclear analytical method, INAA was utilized to determine the presence of Th, U, & K (Eby and Eby, 2014; Lewis, 2005; Kharfi, 2013). The obtained data of U (in μg/g), Th (in μg/g), & K (in %) were then transformed into corresponding radioactivity abundances (Bq.kg^−1^) of ^226^Ra, ^232^Th, & ^40^K by following the formulas presented by IAEA-TEC-DOC (2003).

Hence, targeting the highly susceptible younger aged population, this study presents the NORMs' abundances in the classroom-dust of educational institutions for the first time. The main objectives of this work are to measure the radioactivity contents (Bq.kg^−1^) of ^232^Th, ^226^Ra, & ^40^K in collected dust samples from surface of the classroom’s benches, to evaluate their origin, measured from analytical data, and to evaluate radiological risks for human health.

## 2 Experimental

### 2.1 Study area

**Figure 1:**
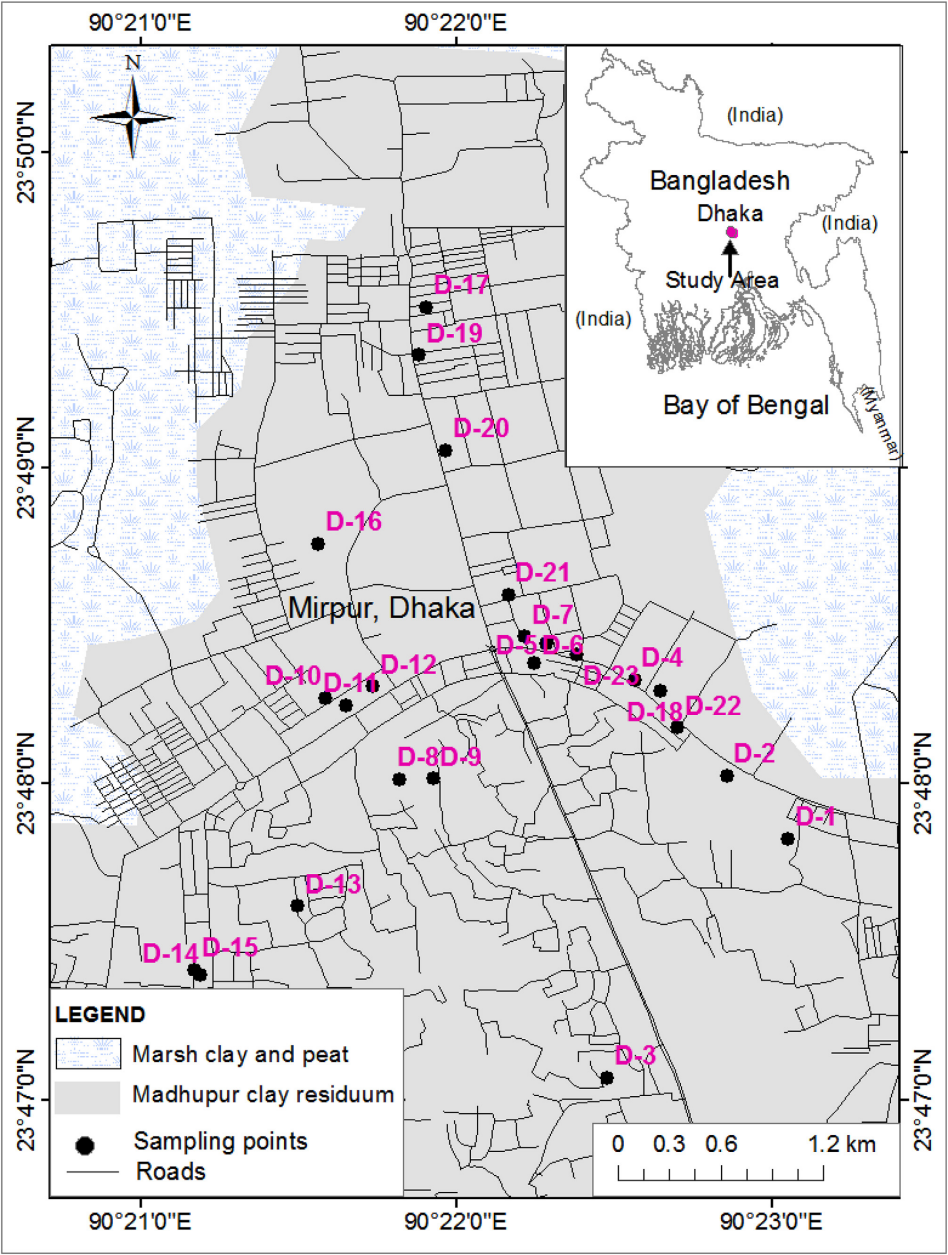
Sampling locations (educational institutions) for the dust samples from central Bangladesh.

The area of interest is positioned in the north-eastern portion of the megacity Dhaka, which is positioned in the central zone of Bangladesh (Figure 1). The overall studied region has extended between 90^0^20′57.25″ E and 90^0^23′25″ E longitude and 23^0^46′52.5″ N to 23^0^50′15″ N latitude. The studied area covers ∼26.2 km^2^ of Dhaka city, whereas the total population of this area is ∼1.25 million according to the last national survey in 2011 (BBS, 2014). A hot and humid tropical weather is dominant in Mirpur, Dhaka and it belongs to the tropical Savanna climate (Kottek et al., 2006). Long-term climate data (1981–2010) indicate that annual temperature varied from 13.1 to 33.8 °C in the Dhaka district. The annual average precipitation is 2085 mm, while 65% of the yearly precipitation takes place during the monsoon (Khatun et al., 2016). Dhaka city is relatively flat land where the topographic elevation varies in the range of 0.5–12m PWD (Public Work Datum) (Rahman et al., 2013).

### 2.2 Sample collection and processing

In Bangladesh, for about 1.5 years (17 March 2020 to 12 September 2021) educational institutions were closed due to the COVID-19 pandemic. During this period, classroom furniture (table, chair, benches) was covered by a layer of dust-particles. Dust samples deposited on the table’s and benches' surface were collected with the aid of a plastic brush in zip-lock polythene bags. From 3 to 5 different classrooms of a single institution dust samples were collected and were mixed together for having a representative composite dust sample. From twenty-three (23) different schools, representative dust samples were collected (Table 1). Collected mass of each dust sample ranged from 15 to 25 g. Collected all classroom-dust samples were then dried by oven (for 24 h at ∼45–50 °C) and were ground to obtain homogeneous sample.

**Table 1:** Samples information of the studied area (Mirpur, Bangladesh) along with their ancillary data.

Sample ID	School Name	Year of Establishment	GPS data	
			Latitude	Longitude
D-1	Monipur High School & College (Br-2)	1969	23°47′49.5″N	90°23′02.9″E
D-2	Rotary School and College	1976	23°48′01.5″N	90°22′51.3″E
D-3	Glory School and College-1	2006	23°47′04.2″N	90°22′28.6″E
D-4	Banaful Adibasi Green Heart College	1976	23°48′19.7″N	90°22′33.8″E
D-5	Mirpur Girls Ideal College	1978	23°48′26.5″N	90°22′17.0″E
D-6	Mirpur Adarsho High School	1966	23°48′23.0″N	90°22′14.8″E
D-7	Senpara Porbota Govt Primary School	1943	23°48′27.9″N	90°22′13.0″E
D-8	Monipur High School and College (Girls)	1969	23°48′00.8″N	90°21′49.1″E
D-9	Monipur Govt Primary School	1966	23°48′00.9″N	90°21′55.6″E
D-10	Sheikh Fazilatunnesa Mohila College	1980	23°48′16.2″N	90°21′35.1″E
D-11	Mirpur College	1970	23°48′14.9″N	90°21′39.1″E
D-12	National Bangla High School	1966	23°48′18.5″N	90°21′44.1″E
D-13	Paikpara Govt Primary School	1986	23°47′36.8″N	90°21′29.9″E
D-14	Wak-Up High School	2000	23°47′23.6″N	90°21′11.3″E
D-15	Wak Up Govt Primary School	1989	23°47′24.6″N	90°21′10.2″E
D-16	Islamia High School	1995	23°48′45.5″N	90°21′33.8″E
D-17	Candour International School	1999	23°49′30.5″N	90°21′54.3″E
D-18	SOS Hermann Gmeiner School and College	1986	23°48′17.7″N	90°22′38.6″E
D-19	Cosmo School	2013	23°49′21.3″N	90°21′52.9″E
D-20	Heed International School	2003	23°49′03.3″N	90°21′57.9″E
D-21	Shaheed Abu Taleb High School	1975	23°48′35.8″N	90°22′09.8″E
D-22	Glory School and College-2	2006	23°48′10.6″N	90°22′42.0″E
D-23	Mirpur English version School and College	2013	23°48′24.6″N	90°22′22.8″E

### 2.3 Instrumental neutron activation analysis

#### 2.3.1 Sample preparation

Approximately 60 mg of each powdered dust sample was weighed in 1 × 1 cm^2^ polyethylene bag, double-packed & heat-sealed. NIST-1633b (coal-fly-ash: standard reference material) was utilized as standard (multi-elemental comparator) whereas reference materials (provided by IAEA) RM-Sl-1 and RM-Soil-7 were utilized as monitoring samples for maintaining the quality of measured data. Along with the studied samples, standard, and control samples, Al-0.1% Au-foil (530RA) was utilized for monitoring the neutron-flux.

#### 2.3.2 Sample irradiation

All collected samples, reference materials, & comparator were put in the irradiation-tube and were irradiated by thermal neutrons (flux: 1.54 × 10^13^ cm^−2^ s^−1^) at 500 kW for 2 h in a 3 MW TRIGA Mark-II research reactor of Bangladesh Atomic Energy Commission (Savar, Dhaka). Following the same experimental process, procedure-blank was also irradiated and necessary corrections were made accordingly (Begum et al., 2021a). Three Au–Al-monitor foils were individually irradiated inserting them at the differential positions (top, middle, & bottom) of the sample stack to monitor neutron flux gradient (Tamim et al., 2016).

#### 2.3.3 Gamma-ray counting, spectrum acquisition, and data calculation

Irradiated samples with systematic decaying were undertaken through γ-ray counting using HPGe-γ-detector (CANBERRA, 40% rel. efficiency, & 1.8 keV resolution) attached with digital γ-spectrometer (ORTEC, DSPEC Jr™) (Khan et al., 2020, 2019b, 2019c). First gamma-ray counting was done in 40 min (after ∼two days of decay) and 2nd gamma-ray counting was done for three (3) hours (after ∼seven days of decay). From the first gamma-ray counting (L1), K & U were identified while Th was identified from the 2nd gamma-ray counting (L2). Genie-2000 (Canberra) and Hypermet PC (version-5.12) were utilized to acquisition of data and analysis of gamma-photo-peak analysis, respectively (Khan et al., 2017, 2018).

#### 2.3.4 Data quality and radioactivity estimation

The accuracy and precision of analytical data (Table 2) were checked by triplicate analysis of Sl-1 & Soil-7. In view of the ranges of analytical uncertainties, determined results of elemental concentrations in control samples were concomitant with the IAEA-certificate values. Precisions (RSDs, %) of this work for the determined elemental concentrations in Soil-7 & Sl-1 ranged from 4.5 to 8.3%. Following are the standard Eqs. (1), (2), and (3) by which elemental concentrations of K (in %), U (in μg/g), & Th (in μg/g) in classroom-dusts were transformed to the accompanied radioactivity abundances (Bq/kg) (Bajoga et al., 2019; IAEA-TEC-DOC, 2003):

(1) 1% K = 313 Bq.kg^−1^^40^K

(2) 1 μg.g^−1^ U = 12.35 Bq.kg^−1^^226^Ra

(3) 1 μg.g^−1^ Th = 4.06 Bq.kg^−1^^232^Th

**Table 2:** Descriptive statistics of elemental abundances of K, Th, and U in repeated analyses (n = 3) of IAEA-RM-Soil-7 (soil) and IAEA-RM-SL-1 (lake sediment) of this study along with the certificate values, detection limits (DL: 3σ), measured radionuclides with their half-lives.

Elements	Unit	IAEA-RM-Soil-7 (soil)						IAEA-RM-SL-1 (lake sediment)						Measured	Half-	Counting	Gamma	DL
		This work			Certificate			This work			Certificate			Radionuclide	life	mood	energy	(3σ)
		Mean	SD	RSD	Value	Min.	Max.	Mean	SD	RSD	Value	Min.	Max.				(keV)	
		(n = 3)	(1σ)	(%)				(n = 3)	(1σ)	(%)								
K	[%]	1.25	0.06	4.8	*1.21*	*1.13*	*1.27*	1.32	0.06	4.5	1.45	1.24	1.66	^42^K	12.36 h	L1	1524.6	0.034
Th	[μg/g]	7.86	0.65	8.3	8.2	6.5	8.7	13.2	0.8	6.1	14	13	15	^233^Pa	27.0 d	L2	312.0	0.07
U	[μg/g]	2.98	0.22	7.4	2.6	2.2	3.3	3.92	0.22	5.6	*4.02*	*3.69*	*4.35*	^239^Np	2.35 d	L1	106.1	0.10

IAEA-RM-Soil-7: Certificate values are taken from the IAEA reference sheet issued in January 2000. IAEA-RM-SL-1: Certificate values for all the elements are taken from the IAEA reference sheet issued in September 1999. All the values are recommended values; except for Italics are information values only. Decay data (here half-life) of the measured radionuclides are taken from Randa et al. (2007).

### 2.4 Radiological indices

#### 2.4.1 Radium equivalent activity (Ra_eq_)

Naturally, radioactivity concentrations of NORMs in the surrounding environmental materials like soil, sediments, or dust are not even**. “**Radium equivalent activity (Ra_eq_)**”** index is applied to eliminate the non-uniform activity of radionuclides (Ravisankar et al., 2015). This can be computed by employing the subsequent Eq. (4):

(4) $${Ra}_{eq}=\left(\frac{A_{Ra}}{370}+\frac{A_{Th}}{259}+\frac{A_{K}}{4810}\right)\times 370$$

where, the radioactivity concentrations of ^232^Th, ^226^Ra, & ^40^K are represented by A_Th_, A_Ra_, and A_K_, respectively. For potential radiological safety appraisal, the maximum permissible value of Ra_eq_ (Isinkaye and Emelue, 2015) were fixed to 370 Bq.kg^−1^.

#### 2.4.2 External & internal hazard indices (H_ex_ & H_in_)

In view to determine the externally exposed radiation (ionizing) doses to specific persons from classroom-dusts, external hazard index (H_ex_) can be computed by Eq. (5). Moreover, the internal hazard index (H_in_) is utilized to assess the radiological hazards posed due to radon & its progenies which can be calculated by using Eq. (6) (Begum et al., 2021b).

(5) $$H_{ex}=\frac{A_{Ra}}{370\;{Bqkg}^{-1}}+\frac{A_{Th}}{259{\;Bqkg}^{-1}}+\frac{A_{k}}{4810\;{Bqkg}^{-1}}$$

(6) $$H_{in}=\frac{A_{Ra}}{185\;{Bqkg}^{-1}}+\frac{A_{Th}}{259\;{Bqkg}^{-1}}+\frac{C_{k}}{4810\;{Bqkg}^{-1}}$$

In which A_Th_, A_Ra_, & A_K_ denote the radioactivity abundances of radionuclides ^232^Th, ^226^Ra, & ^40^K, respectively. According to UNSCEAR, (2000), H_ex_ & H_in_ values should be < 1 for keeping the radiation generated hazard trivial.

#### 2.4.3 Absorbed gamma dose rate (D)

Naturally occurring radioactive nuclides have contributions to the D-values relies upon the radioactivity abundances of NORMs. Moreover, there is an instantaneous association among terrestrial gamma-radiation and activity concentrations of NORMs that once the activity concentration is known later the affiliated exposure dose rate above 1 m air of the ground surface can be computed by using Eq. (7) (Isinkaye and Emelue, 2015; Ravisankar et al., 2015).

(7) D (nGyh^−1^) = 0.462A_Ra_ + 0.604A_Th_ + 0.0417A_k_

Here, the factors demonstrated by UNSCEAR, (2000) for converting the activity contents of ^232^Th (A_Th_), ^226^Ra (A_Ra_), and ^40^K (A_K_) into the dose rate (nGyh^−1^ per Bq.kg^−1^) are 0.604, 0.462, & 0.0417, respectively.

#### 2.4.4 Annual effective dose rate (AEDE)

AEDE can be computed from the D-value by employing the converting factor (CF) of 0.7 Sv/Gy, stay time in the classrooms is 20% of 8760 h.y^−1^ (occupancy factor: OF). As per described above, the AEDE was measured by Eq. (8) (UNSCEAR, 2000).

(8) $$AEDE=D(({nGyh}^{-1})\times 8760(hy^{-1})\times 0.2\times 0.7\times 10^{-6}$$

#### 2.4.5 Gamma representative level index (I_γ_)

I_γ_ can be employed to appraise the natural γ-radiation risk levels from classroom-dusts involved with gamma-emitters. I_γ_ correlated with annual dose-criterion because of excessive γ-radiation by superficial materials and served as like a screening instrument for the sake of determining materials that can cause health concerns. Following Kolo et al. (2015), I_γ_ can be estimated by Eq. (9):

(9) $$I_{\gamma }=\frac{A_{Ra}}{150}+\frac{A_{Th}}{100}+\frac{A_{K}}{1500}$$

#### 2.4.6 Excess lifetime cancer risk (ELCR)

Potential carcinogenic health hazards are represented by measuring the probability of cancer manifestation of individuals for a certain life-time from slope factors (SF) and estimated exposures. ELCR is calculated by employing Eq. (10) (Khandaker et al., 2018).

(10) $$ELCR=AEDE\times A_{lf}\times R_{f}$$

Here, AEDE represents annual effective dose equivalent, A_lf_ denotes the average life-time (70 years) & R_f_ denotes the cancer-risk-factor (Sv^−1^: ICRP, 1990**)** which is considered to be 0.5 × 10^−4^ for the mass public exposures.

## 3 Results and discussion

### 3.1 NORMs' concentrations and distributions

Elemental concentrations of K (in %), U (μg.g^−1^), & Th (μg.g^−1^) in analyzed classroom-dusts along with the estimated radioactivity abundances (in Bq.kg^−1^) are showed in Table 3. The average_n=23_ activity contents of ^232^Th, ^226^Ra, & ^40^K in the classroom-dusts were respectively 43.4, 86.0, and 488 Bq.kg^−1^, which are relatively higher in comparison to corresponding world average values (^232^Th: 30; ^226^Ra: 35; ^40^K: 400 Bq.kg^−1^) (UNSCEAR, 2000). This world average values were considered from the studies carried out before the year of 2000 while this study has complied the data of ^232^Th, ^226^Ra, and ^40^K for surface soils, that were studied after the year of 2000 (Table 4). However, the median_n=23_ values for ^232^Th, and ^40^K of this study were similar to those of concomitant median_n=13_ values of surface soils of Bangladesh (Chowdhury et al., 1999; Hamid et al., 2002; Miah et al., 2012; Miah et al., 1998; Alam et al., 2013; Khatun et al., 2013; Islam et al., 2014; Ferdous et al., 2015; Ehsan et al., 2019; Khan et al., 2019a; Mojumder et al., 2020). However, median values of radioactivity concentration of ^226^Ra in analyzed classroom-dusts is more than two times higher in comparison to the surface soil samples of Bangladesh. Similarly, all the determined primordial radionuclides of this study represented similar trend as the surface soil samples of different countries in the subcontinent (Mishra, 2004; Matiullah et al., 2004; Mandal and Sengupta, 2005; Fatima et al., 2007; Prasad et al., 2008; Shanthi et al., 2010; Badhan and Mehra, 2012; Babai et al., 2013; Dhawal et al., 2013; Sahoo et al., 2016; Pillai et al., 2017; Bramha et al., 2018). Herein, ^226^Ra activities are greater than ^232^Th in the dust samples of this work which is concomitant with the world literature values (Bem et al., 2002; Papp et al., 2002; Faghihi et al., 2011; Flues et al., 2002; Dai et al., 2022; Amin et al., 2008; Arogunjo et al., 2009; Karamanis et al., 2009; Ribeiro et al., 2010; Otansev et al., 2012; Tufan and Bostanci, 2012; Amin et al., 2013; Charro et al., 2013; Papaefthymiou et al., 2013; Cujic et al., 2015; El-Mekawy et al., 2015; Liu et al., 2015; Alzubaidi et al., 2016; Goren et al., 2017; Zhang, 2017) while median_n=23_ value of ^40^K activity in this study was relatively greater than the global median value. Hence, the variation of ^40^K activities generated from differential geochemical settings while the discrepancy of ^226^Ra activity concentrations between the classroom-dusts of this work and the surface soils_n=45_ (Table 4) assumed to be derived from natural or/and the non-natural processes.

**Table 3:** Elemental abundances, their conversion to radioactivity concentrations, and affiliated ratios in the dust samples collected from the classrooms of schools (Mirpur, central Bangladesh) along with their descriptive statistics.

	K		Th		U		Th/U	±	^226^Ra		^232^Th		^40^K		^232^Th/^40^K	±	^226^Ra/^40^K	±	^232^Th/^226^Ra	±
	[%]	±	[μg/g]	±	[μg/g]	±			Bq.kg^−1^	±	Bq.kg^−1^	±	Bq.kg^−1^	±						
D-1	1.42	0.04	11.8	0.1	4.37	0.17	2.71	0.11	54.0	2.1	48.1	0.5	443	12	0.109	0.003	0.122	0.006	0.89	0.04
D-2	1.76	0.04	12.5	0.1	13.4	0.37	0.93	0.03	165.3	4.6	50.6	0.5	552	14	0.092	0.002	0.299	0.011	0.31	0.01
D-3	1.33	0.04	7.17	0.10	8.08	0.26	0.89	0.03	99.8	3.2	29.1	0.4	417	11	0.070	0.002	0.239	0.010	0.29	0.01
D-4	1.93	0.05	17.6	0.1	7.15	0.23	2.46	0.08	88.3	2.9	71.5	0.6	603	15	0.119	0.003	0.146	0.006	0.81	0.03
D-5	1.88	0.04	14.7	0.1	4.12	0.16	3.57	0.14	50.9	2.0	59.7	0.5	587	14	0.102	0.003	0.087	0.004	1.17	0.05
D-6	1.81	0.05	7.93	0.10	7.25	0.24	1.09	0.04	89.5	3.0	32.2	0.4	566	15	0.057	0.002	0.158	0.007	0.36	0.01
D-7	1.88	0.05	11.7	0.1	8.86	0.28	1.32	0.04	109.4	3.4	47.3	0.5	587	15	0.081	0.002	0.186	0.007	0.43	0.01
D-8	1.67	0.04	10.9	0.1	1.98	0.11	5.52	0.30	24.4	1.3	44.3	0.5	522	14	0.085	0.002	0.047	0.003	1.82	0.10
D-9	1.74	0.05	10.7	0.1	4.69	0.21	2.29	0.11	57.9	2.6	43.6	0.5	546	17	0.080	0.003	0.106	0.006	0.75	0.03
D-10	1.83	0.04	11.9	0.1	2.36	0.11	5.04	0.23	29.2	1.3	48.4	0.5	574	13	0.084	0.002	0.051	0.003	1.66	0.08
D-11	1.37	0.04	6.48	0.07	6.74	0.23	0.96	0.04	83.2	2.9	26.3	0.3	428	13	0.061	0.002	0.194	0.009	0.32	0.01
D-12	1.79	0.05	4.56	0.07	7.23	0.24	0.63	0.02	89.3	3.0	18.5	0.3	559	15	0.033	0.001	0.160	0.007	0.21	0.01
D-13	1.23	0.04	9.31	0.10	15.6	0.43	0.60	0.02	193.0	5.3	37.8	0.4	386	12	0.098	0.003	0.500	0.021	0.20	0.01
D-14	1.28	0.04	10.3	0.1	7.21	0.23	1.44	0.05	89.0	2.9	42.0	0.5	400	12	0.105	0.003	0.223	0.010	0.47	0.02
D-15	1.34	0.04	8.94	0.10	7.38	0.25	1.21	0.04	91.2	3.1	36.3	0.4	419	13	0.087	0.003	0.218	0.010	0.40	0.01
D-16	0.72	0.03	5.44	0.07	11.2	0.32	0.49	0.02	138.6	4.0	22.1	0.3	226	9	0.098	0.004	0.613	0.030	0.16	0.01
D-17	1.42	0.04	11.3	0.1	4.64	0.18	2.44	0.10	57.3	2.2	46.0	0.5	445	13	0.103	0.003	0.129	0.006	0.80	0.03
D-18	0.86	0.05	14.6	0.1	8.06	0.28	1.80	0.07	99.6	3.5	59.1	0.6	268	15	0.221	0.013	0.372	0.025	0.59	0.02
D-19	1.36	0.06	15.2	0.1	16.0	0.44	0.95	0.03	197.3	5.4	61.8	0.6	425	18	0.145	0.006	0.464	0.023	0.31	0.01
D-20	1.41	0.06	15.7	0.1	5.00	0.20	3.15	0.13	61.7	2.5	63.9	0.6	440	19	0.145	0.006	0.140	0.008	1.04	0.04
D-21	1.34	0.05	9.51	0.12	1.02	0.07	9.32	0.68	12.6	0.9	38.6	0.5	420	17	0.092	0.004	0.030	0.002	3.06	0.22
D-22	0.42	0.04	3.94	0.07	6.57	0.26	0.60	0.03	81.1	3.2	16.0	0.3	133	11	0.120	0.010	0.610	0.056	0.20	0.01
D-23	1.14	0.06	13.8	0.1	1.16	0.09	11.91	0.92	14.3	1.1	56.0	0.5	356	18	0.157	0.008	0.040	0.004	3.92	0.30
Mean (n = 23)	**1.43**		**10.70**		**6.96**		**2.67**		**86.0**		**43.4**		**448**		**0.102**		**0.223**		**0.88**	
SD (1σ)	0.39		3.67		4.08		2.88		50.4		14.9		122		0.039		0.175		0.95	
RSD (%)	27.3		34.3		58.7		108.2		58.7		34.3		27.3		38.0		78.2		108.2	
Median	1.41		10.91		7.15		1.44		88.3		44.3		440		0.098		0.160		0.47	
Min.	0.42		3.94		1.02		0.49		12.6		16.0		133		0.033		0.030		0.16	
Max.	1.93		17.6		16.0		11.9		197.3		71.5		603		0.221		0.613		3.92

**Table 4:** Radioactivity concentrations of this study are compared with those surface soil samples around the world.

	^226^Ra	^232^Th	^40^K	References/Comments
	Bq.kg^−1^	Bq.kg^−1^	Bq.kg^−1^	
Mean (n = 23)	**86.0**	**43.4**	**448**	This study
SD (1σ)	50.4	14.9	122	
RSD (%)	58.7	34.3	27.3	
Median	88.3	44.3	440	
Min.	12.6	16.0	133	
Max.	197.3	71.5	603	

Literature data				
Surface soils of Bangladesh				
Pabna, Bangladesh	13.4	15.6	202	Ehsan et al. (2019)
Cox’s Bazar, Bangladesh	44.4	69.8	1007	Ahmad and MatiullahKhatibeh (1997)
Gazipur, Bangladesh	66.7	101.6	425	Islam et al. (2014)
Kustia, Bangladesh	6.3	6.6	225	Ehsan et al. (2019)
Natore, Bangladesh	16.5	7.0	143	Ehsan et al. (2019)
Sylhet, Bangladesh	55.3	125.3	498	Miah et al. (2012)
Hobiganj, Bangladesh	11.1	22.0	228	Ferdous et al. (2015)
Chattogram, Bangladesh	35.9	65.5	272	Chowdhury et al. (1999)
Chattogram, Bangladesh	21	40.0	449	Mojumder et al. (2020)
Rajbari, Bangladesh	29	50.9	535	Khatun et al. (2013)
Chattogram, Bangladesh	61	79.7	857	Alam et al. (2013)
Rampal, Bangladesh	34.8	48.9	719	Khan et al. (2019b)
Rangpur, Bangladesh	87	140	1844	Hamid et al. (2002)
Median (n = 13)	34.8	50.9	449	

Surface soils of the Indian subcontinent				
Nasik, India	37.0	69.6	396	Mishra (2004)
Punjab, India	28.6	51.0	570	Badhan and Mehra (2012)
Chennai, India	17.0	55.3	729	Babai et al. (2013)
Kolaghat, India	111.4	140.2	351	Mandal and Sengupta (2005)
Maharashtra, India	45	59.7	218	Dhawal et al. (2013)
Kalpakkam, India	61.7	622.4	416	Bramha et al. (2018)
Bangalore India	26.2	53.1	635	Prasad et al. (2008)
Tamil Nadu, India	58.8	465.2	311	Pillai et al. (2017)
Kanyakumari, India	8.0	22.0	295	Shanthi et al. (2010)
Udisha, India	130	1110	360	Sahoo et al. (2016)
Bhawalpur Pakistan	32.9	53.6	647	Matiullah et al. (2004)
Punjab Pakistan	21.7	31.0	393	Fatima et al. (2007)
Median (n = 12)	35.0	57.5	395	

Global data (surface soils)				
Mawan, South China	199	264	1216	Liu et al. (2015)
Fars province, Iran	26.3	14.9	271	Faghihi et al. (2011)
Baoji, China	32.1	49.8	721	Dai et al. (2022)
Xitulvye, China	49.4	63.5	396	Zhang et al. (2017)
Kapar, Malaysia	86.7	74.3	297	Amin et al. (2013)
Kedah, Malaysia	102.1	134	326	Alzubaidi et al. (2016)
Kangal, Turkey	37.0	17.0	222	Goren et al. (2017)
Kayseri, Turkey	35.5	37.3	430	Otansev et al. (2012)
Egypt	8.6	8.0	252	Amin et al. (2008)
Samsun City, Turkey	19.0	22.0	521	Tufan and Bostanci (2012)
Greece	45.0	32.5	337	Papaefthymiou et al. (2013)
Egypt	14.7	17.1	222	El-Mekawy et al. (2015)
Nigeria	54.5	91.1	287	Arogunjo et al. (2009)
Agios Dimitrios, Greece	26.8	36.8	493	Karamanis et al. (2009)
Serbia	50.7	48.6	560	Cujic et al. (2015)
Douro, Portugal	53.1	46.3	845	Ribeiro et al. (2010)
Figueira, Brazil	133	39.0	233	Flues et al. (2002)
Velilla, Spain	38.7	42.9	445	Charro et al. (2013)
Ajka, Hungary	129	26.9	337	Papp et al. (2002)
Lodz, Poland	16.6	15.7	307	Bem et al. (2002)
Median (n = 20)	41.9	38.1	337	
Median (all, n = 45)	37.0	49.8	396	Radioactivity after 2000
Crust	33.3	42.6	720	Rudnick and Gao (2014)
Threshold values	35	30	400	UNSCEAR, (2000)

**Figure 2:**
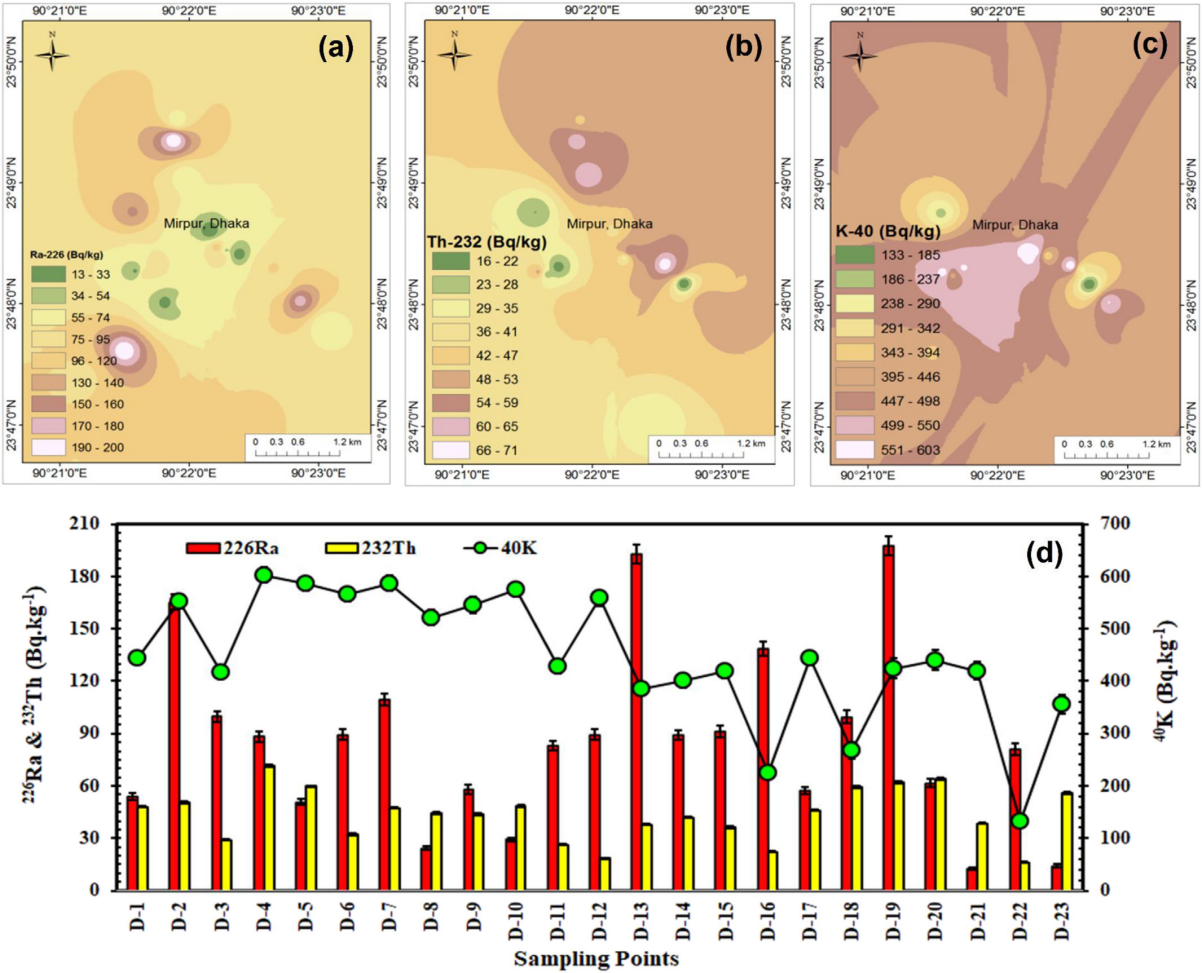
Distributions of primordial radioactive nuclides in central Bangladesh: Spatial distributions of (a) ^226^Ra, (b) ^232^Th, and (c) ^40^K, and (d) comparison of NORMs' distribution among the studied locations.

Radioactivity concentrations of ^232^Th, ^226^Ra, & ^40^K in classroom-dusts ranged 16.0–71.5, 12.6–197.3, and 133–603 Bq.kg^−1^, with the RSDs of 34.3, 58.7, and 27.3%, respectively. Average value of ^226^Ra in dust samples is ∼2.5 times greater compared to the world median value, while, 17.4% (D-8, 10, 21, 23) classroom dusts possess lower ^226^Ra content in comparison to the global data. Considering the U-concentrations in the control samples (Soil-7 & Sl-1), there were no systematic biasness towards the elevated data. Therefore, measured high values of ^226^Ra did not originate from the analytical processes. Furthermore, the individual RSDs (from the counting statistics) for ^226^Ra ranged from 2.7 to 7.7% which were much below than the total RSDs (n = 23). Thus, it can be invoked that distribution of ^226^Ra is inhomogeneous. On the other hand, individual analytical RSDs for ^40^K and ^232^Th range from 2.3 to 8.3% and 0.8–1.9%, respectively, which were also much below than the corresponding total RSDs_n=23_. Hence, the primordial radionuclides in the measured classroom-dusts were inhomogeneously distributed. Inverse distance weighting (IDW) maps (Figure 2a–c) were utilized for visualizing the NORMs distribution and NORMs' radioactivity variations are presented in (Figure 2d). Hot spots (>100 Bq/kg) of ^226^Ra are detected in D-2, 7, 13, 16 and 19, while D-4, 19 and 20 represented moderately higher ^232^Th-radioactivity (>60 Bq/kg). On the other hand, D-4-7, 10, and 12 were identified as hot spots for the distribution of ^40^K (>550 Bq.kg^−1^) and the distribution of ^40^K was relatively homogeneous than those of ^226^Ra & ^232^Th in the study area.

### 3.2 Environmental fractionations of NORMs in classroom-dusts

Elemental concentrations of thorium, uranium, & Th/U-ratio, were 10.7 ± 3.7 (3.94–17.6) μg.g^−1^, 6.96 ± 4.08 (1.02–16.0) μg.g^−1^, and 2.67 (0.49–11.9), respectively. Elemental concentrations of Th and U were relatively higher than those in UCC (Th: 10.5 μg/g; U: 2.7 μg/g; Rudnick and Gao, 2014). However, average Th/U_n=23_ value in this work was noticeably lower compared to the UCC (Th/U = 3.7). Bearing in mind, the activity concentrations of world mean values (UNSCEAR, 2000) for ^226^Ra, ^232^Th, & ^40^K; ^232^Th/^40^K, ^232^Th/^226^Ra, and ^226^Ra/^40^K ratios are respectively 0.075, 0.857, and 0.088. Mean values of ^232^Th/^40^K, ^232^Th/^226^Ra, & ^226^Ra/^40^K ratios in this study are 0.102 ± 0.039, 0.88 ± 0.95, & 0.22 ± 0.18 which vary between 0.03 and 0.22, 0.16–3.92, & 0.03–0.61, respectively (Table 3). Mean value of ^226^Ra/^40^K ratio are noticeably higher, whereas ^232^Th/^40^K and ^232^Th/^226^Ra values are comparable with those of world mean values. RSD_n=23_ of ^232^Th/^40^K value (38%) is the lowermost among the NORMs in classroom-dusts, which means both the radionuclides possess relatively homogeneous distribution. However, considering the RSDs of ^226^Ra/^40^K (78%), and ^232^Th/^226^Ra (108%), it invokes the geoenvironmental fractionation of ^226^Ra in measured classroom dusts.

Alterations of abundances (and corresponding radioactive species) of K, U, & Th, Th/U-ratio in classroom-dusts in comparison to the corresponding UCC-values are inferred to be influenced by progression of weathering of surface soil. Alteration and/or weathering of soil surface (as well as sandy geomaterials) generally can be divided into 2-segments before transformation into the dust particle: (1) Water logging and rain-water fractionate the composition of elements of the soil-surface during wet-season, & (2) Aerodynamic transference of soil from the surface can fractionate the chemical composition of elements in the dry season before resting down as dust-particle.

In the absence of any nuclear installation and agricultural activities, anthropogenic origin(s) of U (corresponding ^226^Ra as well) in the urban environment is unlikely. So, for the dust samples at the site D-8, 10, 21, and 23, the U-enrichment in classroom dust samples and U-depletion can be resulting from the successive natural process. Rain water (with lower pH) usually logged on the soil surface in the rainy season, and flow over the soluble oxyanion-complex [UO_2_^−^] in different places which fractionates ^226^Ra-compositions from the surface-soil that tied loosely. In dry season, when U(≈^226^Ra)-enriched surface soils were exposed to the aerodynamic transference, it results more activity concentrations of ^226^Ra in accompanying dusts. Hence, geo-environmental ruling factors can be the sources of relatively lower mean-Th/U ratio in dusts. On contrary, non-responsive and immobile characteristics of Th towards the redox-condition in the environment (Khan et al., 2021a, 2021b) are preventing the fractionation process of Th from the surface soil in rainy season. Moreover, insoluble precipitates (Th^4+^) and adsorption in organics and/or clays can also be inhibited the mobility of Th (Barnett et al., 2000). Subsequently, Th including lighter clay minerals are taken off by aerodynamic process(es) from the surface soil. Thus, thorium-fractionation probability is much inferior than the probability of uranium-fractionation while the natural/geogenic process(es) are taking place for dust generation, which in turn results in lower Th/U-ratio.

Average elemental concentrations of K (≈^40^K) (average = 1.43 ± 0.39%; 0.42–1.93%) is much lower than the corresponding UCC-value (2.32%). In soils, minerals bearing potassium, e.g, clays, micas, salts, and feldspars are relatively resistant to weathering, while >95% K are being positioned in corresponding minerals' lattice. Nevertheless, during weathering (leaching) and dissolution-alteration these types of minerals released K^+^ ions, which are relocated into the solution of soil and adsorbed in the clays (Kabata-Pendias, 2010; Rachkova et al., 2010). Hence, this adsorption of K^+^ ions in clays took placed until the limit being saturated while the extra K^+^ ions (water-soluble) percolated through the soil of sub-surface. Hence, the loosely-bound soil in the surface become depleted in K in comparison to the corresponding crustal value.

### 3.3 Radiological risks assessment

Human health may have adverse effects owing to the exposure of ionizing radiations from primordial radionuclides (Habib et al., 2019a, 2019b, 2022). Primordial radionuclides are the sources of more than 80% radiation exposures in the ambient environment (UNSCEAR, 2000). Inhalation is quite significant exposure route of radiation compared to the external exposure route through dermal exposure. The gaseous progenies of ^232^Th and ^238^U existing in the ambient environment can cause the internal exposure. The gaseous decay products of ^232^Th and ^238^U are thoron (^220^Rn; half-life: 55.6 s) and radon (^226^Rn; half-life: 3.8 days), respectively which are usually undergone a dilution process with the gases in the atmosphere before entering the human body. Moreover, thoron cannot enter in the human body due to its very short half-life, but radon, after dilution can easily enter into the body by inhalation. However, dust-particles through inhalation can enter into the lungs with accompanying primordial radionuclides, where α-particles with different energies were released from the ^232^Th & ^238^U decay series. In this case, the contribution of thoron cannot be ignored as it emits considerable amount of α-radiation from ^212^Bi (6.1 MeV) and ^212^Po (8.8 MeV). Such alpha radiations with higher energies (even with very low concentration) are capable of breaking the double-strand of DNA, which cause carcinogenic impact on human body.

**Figure 3:**
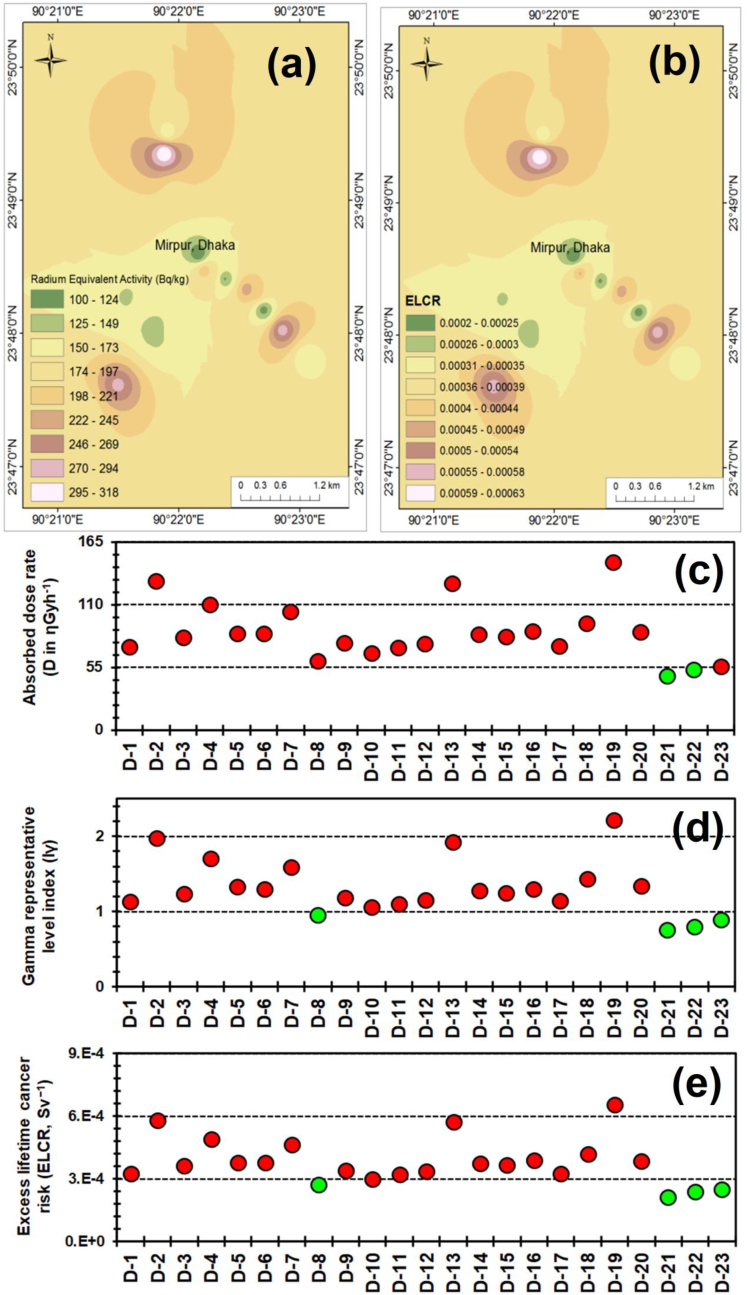
Distributions of radiological indices in central Bangladesh: (a) spatial distributions of radium equivalent activity (Ra_eq_ in Bq.kg^−1^), (b) spatial distributions of excess lifetime cancer risk (ELCR in Sv^−1^), and the spatial variations of (c) Absorbed gamma dose rate (D in nGyh^−1^), (d) Gamma representative level index (I_γ_) and (e) excess lifetime cancer risk (ELCR in Sv^−1^) are distinguished with the threshold values (green: within permissible limit; red: above permissible limit).

Besides the qualitative radiological hazard assessment, radiological indices such as absorbed gamma dose rate (D), radium equivalent activity (Ra_eq_), annual effective dose rate (EAED), external and internal hazard indices (H_ex_ & H_in_), gamma representative level index (I_γ_), & ELCR are utilized to assess the radiological hazards with comprehensible quantitative processes (Table 5) (Figure 3a–e). Ra_eq_ is the collective weighted of the radioactivities of ^226^Ra, ^232^Th, & ^40^K, which is concomitant with the internal & external γ-doses and accompanied with the theory that 4810 Bq.kg^−1^of ^40^K, 370 Bq.kg^−1^ of ^226^Ra, and 259 Bq.kg^−1^ of ^232^Th each of the radionuclides are generating the similar γ-dose rate. In this study, values of Ra_eq_ ranged 100–318 Bq.kg^−1^ with a mean_n=23_ value of 183 ± 54 Bq.kg^−1^, which is much lower in comparison to the threshold value of 370 Bq.kg^−1^. Nevertheless, considering the annual dose-criterion, I_γ_ ranges from 0.75 to 2.22 with average_n=23_ of 1.31 ± 0.37. Only 17.4% (D-8, 21–23) sampling locations have I_γ_ value within permissible value (1.0; Kolo et al., 2015) while the uppermost value of I_γ_ (2.22) was detected at D-19 (Figure 3d).

**Table 5:** Calculated radiological risk indices, e.g., Radium equivalent activity (Ra_eq_), External hazard index (H_ex_), Internal hazard index (H_in_), Absorbed dose rate (D), Annual effective dose rate (AEDE), Gamma representative level index (I_γ_), Excess lifetime cancer risk (ELCR) for the dust samples collected from the classrooms of schools (Mirpur, central Bangladesh).

	Ra_eq_	H_ex_	H_in_	D	AEDE	I_γ_	ELCR
	[Bqkg^−1^]			[ƞGyh^−1^]	[mSvy^−1^]		[Sv^−1^]
D-1	157	0.42	0.57	73.3	0.090	1.14	3.15 × 10^−4^
D-2	280	0.76	1.20	130.8	0.160	1.98	5.61 × 10^−4^
D-3	174	0.47	0.74	81.6	0.100	1.23	3.50 × 10^−4^
D-4	237	0.64	0.88	110.3	0.135	1.71	4.74 × 10^−4^
D-5	181	0.49	0.63	85.0	0.104	1.33	3.65 × 10^−4^
D-6	179	0.48	0.73	85.0	0.104	1.30	3.65 × 10^−4^
D-7	222	0.60	0.90	104.4	0.128	1.59	4.48 × 10^−4^
D-8	128	0.35	0.41	60.6	0.074	0.95	2.60 × 10^−4^
D-9	162	0.44	0.60	76.6	0.094	1.19	3.29 × 10^−4^
D-10	143	0.39	0.46	67.5	0.083	1.06	2.90 × 10^−4^
D-11	154	0.42	0.64	72.6	0.089	1.10	3.12 × 10^−4^
D-12	159	0.43	0.67	76.1	0.093	1.15	3.26 × 10^−4^
D-13	277	0.75	1.27	128.8	0.158	1.92	5.53 × 10^−4^
D-14	180	0.49	0.73	83.9	0.103	1.28	3.60 × 10^−4^
D-15	175	0.47	0.72	82.1	0.101	1.25	3.53 × 10^−4^
D-16	188	0.51	0.88	87.2	0.107	1.30	3.74 × 10^−4^
D-17	157	0.42	0.58	73.6	0.090	1.14	3.16 × 10^−4^
D-18	205	0.55	0.82	93.9	0.115	1.43	4.03 × 10^−4^
D-19	318	0.86	1.39	147.3	0.181	2.22	6.32 × 10^−4^
D-20	187	0.50	0.67	86.5	0.106	1.34	3.71 × 10^−4^
D-21	100	0.27	0.30	47.3	0.058	0.75	2.03 × 10^−4^
D-22	114	0.31	0.53	53.0	0.065	0.79	2.27 × 10^−4^
D-23	122	0.33	0.37	56.2	0.069	0.89	2.41 × 10^−4^
**Mean (n = 23)**	**183**	**0.49**	**0.73**	**85.4**	**0.105**	**1.31**	**3.66 × 10**^**−4**^
SD (1σ)	54	0.15	0.27	25.0	0.031	0.37	1.07 × 10^−4^
RSD (%)	29.8	29.8	37.8	29.3	29.3	28.3	29.3
Median	175	0.47	0.67	82.1	0.101	1.25	3.53 × 10^−4^
Min.	100	0.27	0.30	47.3	0.058	0.75	2.03 × 10^−4^
Max.	318	0.86	1.39	147.3	0.181	2.22	6.32 × 10^−4^
Recommended^a^	370	<1	<1	55	0.46	1.00	2.90 × 10^−4^

a Khan et al. (2022b).

To evaluate the external gamma radiation of classroom-dusts, the absorbed dose rate (D, ƞGyh^−1^) is measured by the quantity of energy radiated from absorbed **i**onizing radiations in unit time and mass of matter. Average D-value for the studied classroom dusts is 85.4 ± 25 ƞGyh−1 which range from 47.3 to 147.3 ƞGyh^−1^. Two sampling sites at D-21, 22 possess the value of D below the permissible value (55 ƞGyh^−1^), while rest of the sample sites exceeded the recommended value (Figure 3c). The value of annual effective dose rate (AEDE) computed by assuming that the students remain in classroom for 20% time of a year. AEDE value ranged between 0.058 and 0.181 mSv/y with the mean value of 0.105 ± 0.031 mSv/y, which are 2.5–7.9 times below the recommended safety level (0.46 mSv/y).

Usually, evaluation of H_ex_ & H_in_ is used to confine the radiation-dose to the permissible dose level of 1 mSv.y^−1^ (ICRP, 1990). Average values of H_in_ & H_ex_ are 0.73 ± 0.27 (range: 0.30–1.39) and 0.49 ± 0.15 (range: 0.27–0.86), respectively, which are below the permissible values. However, 13.0% (D-2, 13, 19) of sampling sites possess dust samples having higher H_in_ value compared to that of recommended value. Furthermore, mean ELCR-value of the classroom-dusts is 3.66 × 10^−4^ Sv^−1^ which is ∼26% higher than recommended value (2.90 × 10^−4^ Sv^−1^). The ELCR-values vary between 2.03 × 10^−4^ and 6.32 × 10^−4^ Sv^−1^, while the sampling sites at D-8, 10, and 21–23 (21.7% of the total samples) were below the permissible limit of ELCR (Figure 3e).

Typical radiological risk indices merely consider the external origins of radionuclides and accompanied ionizing-radiation as well as inhalation of radioactive gaseous progenies (internal exposure). Nevertheless, calculated indices for ionizing-radiation-risk estimation do not take into account of the ingress of radionuclides' sources (classroom-dusts) into the human body (lungs) through respiratory system (by breathing), where radioactive progenies of ^232^Th & ^238^U release α-particles with variable energies. For external radionuclides' sources, effects of such α-particle appear insignificant, as the α-particles can be impeded by dermal hindrance. Nevertheless, internal alpha-particles radiation are not obviously being impeded and can result severe harm to the biological cells. Hence, the estimated radiological hazards denote merely the lowermost limits of radiological health risks for the considered classroom-dusts.

### 3.4 Implications for Bangladesh’s case and potential mitigation approaches

Bangladesh is the 8th-most populous country in the world and more than 165 million people live in an area of 148,460 square kilometres. According to the United Nations (United Nations, World Population Prospects, 2022), the present population of Dhaka metro area in 2022 is about 22,478,000 which is an increase of 3.39% from 2021. Bangladesh is on a way to graduating from the UN’s Least Developed Countries (LDC) list in 2026. Keeping this view, Bangladesh has taken a number of development projects specially on the infrastructural side like the metro-rail project, flyover, and under-pass. Moreover, a number of industries like textiles, garments, and pharmaceutical companies are also located in Dhaka city. Hence, the produced dust from these development works and transportation can affect the environment adversely. The mean concentrations (in Bq.kg^−1^) of ^226^Ra (86.0), ^232^Th (43.4), and ^40^K (488) in the classroom-dusts are comparatively greater in comparison to the corresponding world average values (UNSCEAR, 2000) which may cause the carcinogenic and non-carcinogenic health risks. Ahmad (2008) demonstrated that school children in Dhaka have been suffering increased respiratory difficulties due to the elevated level of particulate matter in the ambient air. According to Sakib (2021), minimum 200,000 people could die in Bangladesh due to respiratory diseases and long-term exposure to highly contaminated air. The mean concentration of ^226^Ra in Bangladesh from different literature (n∼13) was 34.8 Bq.kg^−1^ (Table 4) but this study found the mean concentration of ^226^Ra in Dhaka city is 86.0 Bq.kg^−1^ which may cause serious health issues for the residents, specifically the school going students of Dhaka city. Both children and adults are died due to the adverse effects of air pollution and/or other diseases linked to it have risen sharply in the country. The AQI reported that the average PM_2.5_ concentrations in Bangladesh is 15.4 times higher compared to the WHO annual air quality guideline (AQI Report, 2021) value 2021 and about 13–22% of deaths in this area are associated with the air pollution exposure. So, the air containing dust adversely affects the environment by settling down the indoor establishments. This study presents the worst-case scenario by selecting roadside educational institutions residing in highly dense demographic areas of Dhaka city, and a greater number of students have been considered. Obtained data from this study represent that produced dust from construction work and movement of transportation contained radioactive nuclides which have significant radiological health hazards for school-going children. Throughout this study ‘potential health risk impacts due to the NORMs in dust particles’ are brought into the daylight for a developing country like Bangladesh.

NORMs are the inherent geochemical constituents of the dust samples. So, to reduce the radiological health risks originating from the dust particles, utter concentration should be given in reducing dust production. Vehicular and construction-related emissions are a significant source of dust production which should be reduced. In doing so, dispersion of dust particles may be reduced by spraying water in the premises of schools and nearby roads, especially during the dry season (Wallace and Cheung, 2013). Additionally, demolitions of older establishments and construction works should be covered to reduce dust dispersion. Nevertheless, construction personnel are mostly unaware of the carcinogenic health impacts of dust (Wu et al., 2016). Therefore, one of the first steps to achieving successful dust pollution control is to improve public awareness regarding this issue (Lahiri et al., 2005). Relocations of educational institutions far from high traffic areas to more green areas, could be another approach to reduce these health risks. However, such relocation may not be economically viable. Herein, the arrangement of air ventilation and filtration devices, monitoring air quality by low-cost devices, etcetera can be installed to reduce the exposure of students to dust particles (Oliveira et al., 2019). Additionally, personal health consciousness, like wearing a mask could be another effective measure to elevate the radiological health risks originating from the dust. Furthermore, tree plantation in and around educational institutions can reduce the entrance of dust particles into the classrooms (Zhang et al., 2017; Serbula et al., 2012). In such cases, leafy trees can act as natural dust filters. Finally, proper environmental regulations should be strictly monitored to reduce the industrial emissions of dust materials (Wu et al., 2016; Zhang et al., 2015, 2017).

## 4 Conclusion

For the first time, this work discovered the presence of NORMs-concentrations in the classroom-dusts collected from various educational institutions of central part of Bangladesh. The mean NORMs concentrations in the dust samples are higher in comparison to the associate world mean value, especially the activity of ^226^Ra is ∼2.5 times higher. The NORMs' radioactivities of this present study are compared to the activity of surface soils of different countries round the world, which correspondingly unveiled the enrichment of naturally occurring primordial radionuclides in the classroom-dusts relative to the pedosphere surface. Variation of local-geochemistry, variable solubility-based elemental fractionations, leaching, chemical weathering, adsorption, followed by transference of aerodynamic particles control the concentration of NORMs' abundances in the studied classroom-dusts.

The typical measures of radiological indices evaluate the radiological risks. The average values of H_ex_, H_in_, Ra_eq_, and AEDE were within the corresponding permissible limits, whereas the mean values of I_γ_, D, and ELCR were surpassed the recommended safety limits significantly. Albeit the present quantitative approach demonstrated merely the lowermost levels of radiological health risks, overlooking the issue of NORMs' comprising dust particle’s ingress into the respiratory system by breathing. However, COVID-19 pandemic compelled us to wear mask, which might have a positive impact on hindering the ingress of dust particles into the respiratory systems. Herein, this study can draw the public awareness and the policy maker’s attention regarding the radiological hazards of dust on the school going students. This work eventually invokes the construction works and urbanization processes with limited production of dust materials.

## Declarations

### Author contribution statement

Md. Joynal Abedin: Performed the experiments; Analyzed and interpreted the data; Wrote the paper.

Rahat Khan: Conceived and designed the experiments; Contributed reagents, materials, analysis tools or data; Wrote the paper.

### Funding statement

This research did not receive any specific grant from funding agencies in the public, commercial, or not-for-profit sectors.

### Data availability statement

Data included in article/supplementary material/referenced in article.

### Declaration of interest’s statement

The authors declare no conflict of interest.

### Additional information

No additional information is available for this paper.
